# Experiences of drug use and ageing: health, quality of life, relationship and service implications

**DOI:** 10.1111/j.1365-2648.2010.05378.x

**Published:** 2010-09

**Authors:** Brenda Roe, Caryl Beynon, Lucy Pickering, Paul Duffy

**Affiliations:** 1 Brenda Roe PhD RN RHV Professor of Health Research Evidence-based Practice Research Centre, Faculty of Health, Edge Hill UniversityUK; 2 Caryl Beynon BSc PhD Reader in Epidemiology of Substance Use Centre for Public Health, Faculty of Health, Liverpool John Moores UniversityUK; 3 Lucy Pickering BA MA PhD Research Fellow Health and Social Care, Faculty of Health, Oxford Brookes UniversityUK; 4 Paul Duffy BSc Research Manager Centre for Public Health, Faculty of Health, Liverpool John Moores UniversityUK

**Keywords:** ageing, drug use, health, nursing, quality of life, relationships, service use

## Abstract

roe b., beynon c., pickering l. & duffy p. (2010)Experiences of drug use and ageing: health, quality of life, relationship and service implications. *Journal of Advanced Nursing***66**(9), 1968–1979.

**Aim:**

This paper is a report of an exploration of older people’s experiences of substance use in the context of ageing, and its impact on health, quality of life, relationships and service use.

**Background:**

Use of illicit drugs by older people is a neglected policy, research and service provision and is generally perceived as a lifestyle of younger populations.

**Method:**

A convenience sample of 11 people aged 49–61 years (mean 57 years) in contact with voluntary sector drug treatment services participated in qualitative semi-structured tape-recorded interviews and thematic content analysis was performed. The data were collected in 2008.

**Findings:**

Drug use can have negative impacts on health status, quality of life, family relationships and social networks that accrue with age. Participants were identified as early or later onset users of drugs due to the impact of life events and relationships. A range of substances had been used currently and throughout their lives, with no single gateway drug identified as a prelude to personal drug careers. Life review and reflection were common, in keeping with ageing populations, along with regret of ever having started to use drugs. Living alone and their accommodation made them more susceptible to social isolation, and they reported experiences of death and dying of their contemporaries and family members earlier than usual in the life course.

**Conclusion:**

Older people who continue to use drugs and require the support of services for treatment and care are an important emerging population and their specific needs should recognized.

**What is already known about this topic**

- It is estimated that the numbers of people aged 50 years and above requiring treatment for drug or alcohol problems will increase.
- Services specifically for older people who misuse drugs are not widely available, and access by this ageing population may not be facilitated.
- Use of illicit drugs by older people is neglected in policy, research and service provision and is generally perceived as a lifestyle of younger populations.

**What this paper adds**

- Older people who have used or continue to use problematic or illicit drugs were classified as early or later onset users, with no single gateway drug initiating their drug careers.
- These people are a vulnerable group who have impaired health status, chronic illness conditions, health needs and poorer quality of life due to drug use, addiction and life experiences, and require health and social care and voluntary sector services.
- Relationships, social networks and support for this convenience sample were restricted due to their lifestyle and contributed to their vulnerability and living alone potential social isolation.

**Implications for policy and/or practice**

- Further research with older people who continue to use drugs is warranted in order to understand the consequences for individuals and their families and to inform policy, service provision and treatment.
- Health and social care professionals working with older people should enquire about and take account of drug use, identify specific needs and tailor services, treatment and care to meet their individual needs.
- Older people who have used or continue to use drugs may have a role in education of younger populations to prevent drug use.

## Introduction

With populations ageing, it is estimated that people aged 50 years and over requiring treatment for drug or alcohol problems will also increase. In the United States of America (USA) this number is projected to rise from 1·7 million people in 2000 to 4·4 million by 2020 (Gfroerer *et al.* 2003), and in Europe it has been suggested that people aged 65 years and over requiring treatment will double between 2001 and 2020 (EMCDDA 2008). In the United Kingdom (UK), the proportion of problematic drug users aged 50 and above in contact with drug treatment services in Cheshire and Merseyside has increased statistically significantly from 1998 to 2005 from 1·5% to 3·6% for men and from 1·9% to 3·2% for women (*P* < 0·001; Beynon *et al.* 2007). Problematic drug use is defined in the UK as use of opiates and/or crack cocaine (Home Office 2008). However, recent reviews suggest that services specifically for older people misusing drugs in the UK are not widely available or accessed by them, and that diagnosis of drug and substance misuse among this population is missed and access to services and treatment not provided (Crome & Bloor 2005a, 2005b, 2006).

## Background

Substance misuse among older people is a neglected but treatable issue (Gossop & Moos 2008). Older drug users have been reported as having increased morbidity compared to the general population (Hser *et al.* 2004), and being more likely to experience loneliness, stress and fear of victimization (Levy & Anderson 2005). Abuse of alcohol and prescription drugs among older people has been investigated (Hser *et al.* 2001, Gilhooly 2005). Use of illicit drugs by older people has been largely neglected in policy, research and service provision and is generally perceived as behaviour of younger populations.

## The study

### Aim

The aim of the study was to explore older people’s experiences of substance use in the context of ageing, and its impact on health, quality of life, relationships and service use.

### Design

An exploratory qualitative study was undertaken using semi-structured interviews with prompts.

### Participants

A convenience sample was recruited from a voluntary sector drugs treatment service in the North West of England. Staff informed clients aged 50 years and above who currently used drugs about the study, and if they were interested gave them an information sheet. The names and contact details of clients willing to participate were passed on to the research team. Contact was made with potential participants and a convenient time and location that assured privacy and safety for the interview was arranged. Prior to interview participants were re-issued with the project information sheet and consent form, and received an oral explanation of the project followed by time for questions. The final sample size recruited was determined by thematic saturation of the data, with no new themes being identified (Morse 1995).

## Data collection

Tape-recorded semi-structured interviews with prompts were conducted with individual participants and lasted up to 1 hour. Data were collected between January and February 2008. Information was collected on:

1. History and type of substance use
2. Health, well being and quality of life
3. Lifestyle, employment and finance
4. Family, relationships and social networks
5. Use of health and social services
6. Issues across their earlier and later life

### Ethical considerations

The study was approved by the appropriate ethics committee. Permission to use anonymous quotes was obtained as part of the informed consent process. Each participant was given a £10 shopping voucher for local high street shops in recognition of their participation in the project.

### Data analysis

Tapes were transcribed and initially checked for accuracy. Each member of the research team then independently undertook a preliminary content analysis to identify broad themes. These were discussed and agreed, and then each transcript was re-read by individual members and more detailed themes and sub-themes were identified. Further discussion and agreement of themes and sub-themes was undertaken and saturation of data agreed. Individual narratives and life stories have been compiled (Denzin & Lincoln 1994), and quotes used to illustrate findings for each of the themes identified.

### Reliability and validity

The first three interviews undertaken constituted pilot work. No changes were required in the interview schedule and prompts. Initially people aged 60 years and over were to be recruited, but sufficient numbers proved difficult to attract; therefore the age range was reduced to 49 years and above to maximize recruitment. Following content analysis, no difference in the themes and sub-themes were noted for the pilot interviews and data from all the interviews were included in the findings. Validity was assured during the interviews by using lines of questioning to verify accuracy and consistency of responses. Reliability of the analysis was assured by members of the team reading the transcripts, followed by discussion and agreement of the themes and sub-themes identified until saturation was obtained.

## Findings

Interviews were conducted with a sample of 11 participants aged 49 years and over (nine men and two women; mean age 57 years, range 49–61 years). All participants were single, with five having been previously married but now divorced and eight living alone. Two lived with a male friend who acted as a carer, one lived next door to a friend/male carer, and another shared accommodation with other drug using ‘mates’. Their accommodation varied, with some living in a hostel, their own homes (council house, flat or housing association bedsit), a care home or caravan. Interviews took place in a general practice surgery (*n* = 2), at home (*n* = 2), in the care home (*n* = 1) or at the drug service facility (*n* = 6).

The findings reported in this paper relate aspects of ageing to drug careers, health status and quality of life, relationships, social networks and service use.

### Drug careers: past and present

Participants were categorized into two types of user: ‘*early onset’* or ‘*later onset’* problematic drug use. Early onset drug use commenced for most of those interviewed during adolescence or early adulthood as a consequence of recreational use, experimentation, escape or part of the ‘hippie era’, or was triggered by child abuse and/or a parent dying, as this typical quote indicates:

Just bush (cannabis) (17 years) and then from there it went on to other things, y’ know, until I got on to the hard stuff. I was about 21. (Man, 61 years)

‘*Late onset’* problematic use of illicit drugs began later in the life course for some due to stressful life events such as divorce or death, or because of close personal relationships with a ‘drug user’, evidenced by this man talking about his partner:

She’s been using for another 6 months, so I was absolutely devastated. So I took them, thinking that the shock of how much she loved me, seeing me take drugs would stop her taking them. Y’know, that was how naïve I was. And subsequently I ended up with a habit. (Man, 54 years), started using 6 years previously.

Table 1 illustrates timelines of participants’ commencement of drug use and their past and present drug careers. No single drug was identified as a gateway into their personal drug use, and a variety had been used:

**Table 1:** Timelines of life events and patterns of drug use for older people interviewed

Participant	Drug use and key life events	1940	1950	1960	1970	1980	1990	2000
01Male, 61 years	Drug use			First cannabis and acid. Later heroin, cocaine and amphetamine		Abstinent abroad, drug taking on visits home	Meet old friends, drug use continues. Stops drinking	At interview, on methadone
	Life events	Born		10 years in London, drug taking. 18 months in prison for drug related offences		10 years working in Middle East	Moved back to Merseyside	Suffers stroke
02Male, 54 years	Drug use				Amphetamine and acid at weekends –‘non-drug users’. Alcohol use	Nothing for about 20 years		Starts heroin and crack. At interview, methadone and a little alcohol
	Life events		Born		Engineer, property developer: successful business man			Gets married to a drug user. Prison. Hepatitis C. 9 months homeless
03Male, 56 years	Drug use			First cannabis. Later heroin because no cannabis around. 10-year break – starts again because of accident		Starts using methadone		Starts using cocaine. At interview; on methadone
	Life events		Born		Accident means he can no longer work			
04Male, 56 years	Drug use				First cannabis, acid and amphetamine. Opium	Starts using heroin, this becomes heavy when relationship ends	Starts using methadone	Recommences heavy heroin use following death of partner
	Life events		Born			Intermittent heroin use and work. Long-term relationship ends	Hepatitis C	Partner dies. Diagnosed with manic depression (previously misdiagnosed as depression)
05Male, 61 years	Drug use			First amphetamine, morphine and hydrochloride – drugs taken when available. Injects heroin before smoking it	White heroin, cannabis and occasionally speed and organic cocaine	Moves to brown heroin when white no longer on sale. Had 7 years off heroin but in this time, drank heavily. Major relapse after relationship ends		No drug use for time while in Holland, started using again on return when home
	Life events	Born		Starts training as a nurse	Moves to London	Relationship ends		
06Male, 58 years	Drug use			First amphetamine. Is prescribed anti-depressants, heroin, cannabis	Cannabis, cocaine and heroin. Drug use increases when Mum dies	Injects cocaine, heroin. Barbiturates		Cocaine and heroin. At interview, on methadone
	Life events		Born	Detention and borstalAbusive father	Prison sentences. Moves around. Mum dies	Develops agoraphobia		
07Female, 49 years	Drug use					Amphetamine. Cannabis. Heroin		Smokes cocaine (previously injected it). At interview, on methadone
	Life events		Born		Has daughter	Two long-term partners die. Resumes friendship with old drug taking friends and starts injecting. Hospitalized with abscess		
08Male, 61 years	Drug use			Amphetamine, purple hearts, cannabis, speedballs, mushrooms, LSD. Drug use increases when Dad dies. Injects heroin	Benzodiazepines and injects cyclazine. On and off heroin			At interview, on methadone scripts Benzodiazepines occasionally
	Life events	Born		Dad dies. Prison terms. Gets hepatitis. Diabetes				
09Male, 56 years	Drug use			Cannabis. Amphetamine	Initially uses heroin in order to sleep so he can work		Methadone	At interview, on methadone. Injects heroin. Alcohol
	Life events		Born	Drug use escalates after friend dies and after loosing his flat				
10Male, 58 years	Drug use			Amphetamine. Heroin	Cannabis. Crack. Heroin on and off	Heroin		At interview, on methadone. Heroin
	Life events		Born		Travels around through work	Prison		
11Female, Aged 61 years	Drug use				Starts drinking following divorce		Heroin and stops drinking. 10 years of not using heroin ends when mother dies.	At interview, on methadone
	Life events	Born		Works	Divorce. Prison sentences		Mother dies. Cirrhosis	

Born, born in that decade; LSD, lysergic acid diethylamide.

None of us had seen the weed or anything ‘cos we were in pills and everything. And when I went away and then got introduce to weed, y’know, and people says, ‘Oh no, you do it the other way. You do it any way you want to, whatever comes first’. (Man, 58 years)

Consequently, a variety drugs was used first, such as amphetamines, cannabis, lysergic acid diethylamide (LSD), ‘magic mushrooms’ (containing psilocybin), morphine, heroin, speedballs (heroin plus, usually, cocaine), and people used drugs according to availability and personal choice based on their effects. Alcohol use and tobacco smoking also featured in their drug use repertoires (Table 1).

Characteristic features of drug taking patterns across their lives included drug/s of choice; periods of reduction, cessation and abstinence or cycles of relapse, maintenance and substitution of drugs and alcohol; and near continuous and even escalating consumption, for example of heroin as well as methadone and other drugs (Table 1). A characteristic of participants’ ageing, and perhaps the influence of drug treatment services, was that all were trying to ‘use’ responsibly and to maintain their personal safety based on previous experiences. Such experiences included their own and others’ overdoses, accidental and intentional deaths of ‘drug-using mates’, hospitalization, ill-health and unsafe personal situations in which they found themselves:

I learnt a bit of sense. I learnt sense. Still on the gear, know what I mean? But I learnt a bit more. ‘Cos I used to just get stoned and walk round, y’know – just fall over and all, y’know, the way everybody do (they’re young?). But now I just take it … don’t annoy nobody. Nobody can say anything. (Man, 56 years)

and

So I’ve got to be very careful – my body’s not what it was. Occasionally I can get carried away and I’ll be feeling, because I’m feeling young inside, I’ll be feeling young outside, which is just not so. These days I’ve got a handle on that. If I’ve been drinking a lot, I won’t smoke. Even if I’m not feeling too good, I’ll leave it until the alcohol’s worn off some to make sure that I’m not going to go asleep and not wake up. (Man, 61 years)

### Health and quality of life

Participants generally recognized that their drug use was having detrimental effects on their health. It had resulted in a range of chronic and life-threatening conditions which related to their physical and mental health, resulting in hospitalization and the need for ongoing treatment. Their health conditions included circulatory problems, such as deep vein thrombosis, injection site ulcers, stroke, respiratory problems, pneumonia, breathlessness, and conditions such as diabetes, hepatitis and liver cirrhosis. Malnutrition, weight loss, obesity and impaired mobility were also consequences. Impacts on their mental health included memory loss, paranoia and changed mood states, with anxiety and anger being common. Accidental injury due to falls and drug overdose also featured.

Changes in health status seemed cumulative consequences of drug use. One man aged 61 years said that his health had ‘dramatically deteriorated’ and, despite their survival and resilience, there did seem to be an accelerated experience of health changes with ageing. Some put these down to ageing and others to drug use or a combination of the two:

I’ll arrange to see somebody, I’ll forget. It happens all the time, it’s happening more and more regular as I’m getting older, my short memory loss. I can only put it down to just, down to drug abuse and drink abuse, really. I can’t put it down to anything else. (Man, 61 years)

Participants reflected on their lives and drug use over time. This life review made them remark on their survival, despite so many friends and acquaintances having died prematurely, and their own resilience. Death and dying was a common feature for all participants confronting their own or other people’s situations, and seemed to be a specific accelerated feature of ageing for them. A man described using drugs and its associated lifestyle and culture as ‘disgusting and squalid’ (61 years). A lifestyle of continued drug use and ageing was illustrated by one man, who said:

The more I seem to get older, the more it seems to go worse. At 56 now, I shouldn’t be doing this. I shouldn’t be going out grafting (*illegal work, usually acquisitive crime*) and then running round like a 19-year-old scally (*scallywag*) looking for heroin and coke. I shouldn’t be doing all this. Like I shouldn’t even be on meth (*methadone*) now. Don’t know, it’s just craziness. It’s madness. (Man, 56 years)

All wished they had not started to use drugs, and some would advise younger contemporaries not to start or to quit, but commented that they did not seem to listen to their wisdom of experience. Drug addiction is powerful and hard to quit, with one man reporting that he did not want to grow old but knew that he would never give up drugs as this is too difficult. A small minority of participants aspired to abstain from drug use, and were abstinent or were reducing their drug use in order to become eligible for a detoxification programme and abstinence supported by drug treatment services. They expressed goals and life plans for the future. A majority were continuing to use drugs and were maintained by methadone replacement provided by primary health care or voluntary drug treatment services. Only a few reported that their drug use had increased despite their advancing age, and involved heroin as well as methadone, and they put this down to their living accommodation, living with fellow drug users, relationships and consequent lifestyles. They did, however, try to ‘use’ drugs safely and not put themselves at undue risk, which they might not have considered when they were younger.

### Relationships and social networks

All participants were single or divorced, and loss of relationships with family members, spouses or partners and with their children due to divorce, death or drug use was a common feature. For a majority, drug use coincided with chaotic and non-sustaining relationships and lifestyles, with some also experiencing repeated imprisonment. Three men now relied on male carers who were also drug users for help and support with daily living. For others, pets provided key relationships which gave structure to their days, responsibility and companionship.

Loss of key relationships and social networks may have been an amplified and accelerated feature of ageing for participants as a consequence of their drug use over the life course. Confronting death and dying of oneself and others is a feature of ageing, but for this group of people earlier and accelerated experiences of loss, death and dying and their magnitude were marked. Loss of access to children and deaths of friends were described as heartbreaking. This was illustrated by a man who had experienced the early death of his father, which precipitated his drug career (at aged 14 years), the death of a baby son, and deaths of other drug-using friends and acquaintances:

But there were people I would not sell to ‘cos I knew they’d end up dead. Y’know, guys who’d just hit up and hit up and hit up until there’s nothing left of them.Yeah. I found a few dead bodies as well (friends)… I went to theirs and (he) was in the chair. I just walked in the door and it was open. Oh stink, y’know, the smell in there was terrible, and I kicked a chair – he had this weird chair…And it went... he were dead, stone cold dead. (She) was upstairs full of maggots, she was a terrible state. I just called the police and they got rid of the bodies, but I had to go to a coroner’s court just say that I found them. The downside to drugs is bad… One of my brothers was murdered and my other brother, he’s got a family and I sometimes wonder what it would have been like if I’d had… I have kids but don’t now where they are. I mean, that’s the way life is. (Man, 61 years)

This man attributed his own survival to frequently being in prison and the resulting enforced abstinence from drugs. He appreciated the close support of his remaining siblings and believed that his mother would have been proud that he has survived. He reflected that he would not want to be a burden on his family and would rather die. Another man, aged 58 years, said that he thought about dying every night as most of his friends were dead, and he continued to use drugs. He stated, ‘To die in your sleep is best’. While for many the loss of relationships with parents, siblings, partners, spouses and children was a feature, for a few, like the man above, family support was vital and enduring. One woman (aged 61 years) reported that her family did not want to know her while she was using heroin, but now that she was clean her family ‘are all around me’ and ‘I feel more happier’. She was not depressed: ‘Now I am off the two of them (alcohol and heroin) I am happy as a lark’ and ‘I feel better now than when I was younger’.

Chaotic relationships were a feature and related to past and current drug use. Some participants described other users as ‘drug buddies’, ‘part of a fraternity’ and said that ‘users mix with users’; however, they described them as not ‘true friends’. Some stated that they did not have any friends: ‘I’ve got no friends. I don’t associate with nobody. I only had two – one’s dead and the other I haven’t seen for 20 years’ (man, aged 58 years). Loss of supportive relationships, whether familial or friendship, deteriorating health and quality of life and living circumstances made them increasingly vulnerable and potentially socially isolated.

### Service use

All participants were in contact with voluntary sector drug services, which reflected how the sample was recruited. They were generally positive about the care and support they received from drug treatment services, which largely met their needs. They also described other formal services they had received, such as primary health care, general practice or dental care, hospital and social services. In contrast, their experiences of these were mixed, with examples of less-than-favourable care due to their being drug users and stigma or prejudice of healthcare professionals, but with some instances of compassionate care and acknowledgement of their drug-using status.

The age range of the sample was 49–61 years, with some having been in contact with drug treatment services for over 20 years. A few participants revealed that they knew of contemporaries aged in their late 60s and early 70s who continued to smoke or inject heroin. It is unknown whether they were receiving drug treatment services. Therefore health and drug services are providing care to an increasing population of ageing drug users, although there may be cohorts of older users who are not receiving services and have unmet needs.

The majority of participants were having methadone replacement/maintenance treatment, with a minority now abstinent or aiming for abstinence. They were generally positive about the specific drug treatment services and care provided, staff attitudes and relationships, and reported benefits. One woman described how bereavement counselling had been particularly helpful, from which this specific population could particularly benefit. In contrast, there were mixed reports of their own or friends’ experiences of hospital and primary health care, which made some fearful of going into hospital:

I won’t go into hospital – my doctor goes mad. I went in and, ‘cos you tell them you’re on methadone, they come in one night… and they put it in one of them big syringes and squirted into my mouth in front of everybody. Makes you feel that big. You know, they shouldn’t do that, they shouldn’t do that at all. (Man, aged 58 years).

Others described instances of care and compassion by nurses which helped them recover and regain weight with improved nutrition. Services with tailored treatments based on compassion and care are required and feasible. There were some reported difficulties in accessing residential detoxification programmes, and preferences for individual as opposed to group-based treatment sessions, which illustrates the need for individualized and targeted care. Participants acknowledged that drug services were more widely available and accessible compared with when they were younger, and this was seen not only as positive but might have made a difference if they had been available earlier in their lives. It was thought that younger users, if they accessed services now, could avoid a lifetime of drug abuse and detrimental consequences:

You know, years ago we didn’t have the help they’ve got now. You know, there’s a lot of help now but the kids won’t take it. They don’t take it. As I says, in our day it was just get on with it – nobody wanted to know you. You were a disgrace. But now everybody’s on it, so you can’t win can you? (Man, aged 58 years)

Their advice to younger people would be to ‘keep off and not use drugs’. Ageing drug users and former drug users may have a role to play in service provision and prevention, based on their life experiences.

A further consideration for service provision is that older drug users may have different requirements and needs compared to younger users, and more specific tailored services are required:

It’s made me extremely depressed, in as much as the few that were left are people I could talk to, and at least there’s consolation in company if it’s good company. And the way it is at the moment the only company I could find if I wanted to, apart from the likes of (D), who’s an exception to the rule…would be people who are younger and on that totally different scene, and, like I say, I have nothing in common with them. I don’t want to sit round a room talking about who sells the biggest bags, I don’t want to sit round rooms talking about who sells the best crack, and that’s the sum total of their conversations. And who stole off who, who didn’t pay the fiver back the other day for the heroin he took from so and so.’ (Man, aged 61 years)

## Discussion

### Study limitations

This was an exploratory qualitative study with a convenience sample of ageing predominantly male illicit drug users, and has provided information on issues of drug career, health status, quality of life, relationships, social networks and service use related to ageing within one geographical area of the UK and can help to inform future research and service development. This is an emerging area of investigation as an increasing number of drug users are surviving into older age (Levy & Anderson 2005, Beynon *et al.* 2007, Gossop & Moos 2008) and require the use of public and voluntary services to remain supported and living in the community. However, the study had a small sample, reflecting the lower prevalence in this ageing cohort and the difficulty of access and recruitment, and did not include affluent ageing drug users who could afford private treatment. Their experiences of ageing, drug career, needs and service use could differ. The study did not include older drug users who did not access support from drug treatment services, and they may also have different experiences and needs. A larger, longitudinal study with ageing cohorts representative of drug career, gender and social status from wider geographical areas is warranted.

### Drug career and ageing

Ageing drug users could be classified into *early* and *later onset users* and a variety of drugs served as a *gateway* to drug use, as opposed to the general public perception that a particular drug or drugs or a pattern of drug-taking is responsible. Public health initiatives in the UK have largely targeted younger users (*early onset users*), focusing on preventing younger people starting drugs, harm reduction for individuals and communities, crime reduction, improved access to treatment and reduction of drug supply (Drug Strategy Directorate 1998, 2002; National Institute on Drug Abuse 2003, Levy & Anderson 2005, Gossop *et al.* 2006, Home Office 2008). The common belief that drug use discontinues in middle age due to illness, death, voluntary cessation or for other reasons is simplistic and may not reflect real ageing and cohort effects (Gilhooly 2005, Levy & Anderson 2005). Our findings concur with those in other studies that some people are *late onset users* as a result of life events and relationships, rather than early youthful escapism or experimentation (Roberts 1999, Johnson & Sterk 2003, Levy & Anderson 2005). There is emerging evidence that drug use continues in old age and research is needed to understand the issues and consequences for older people and their families, and the implications for public services (Phillips & Katz 2001, Crome & Bloor 2005a, 2005b, 2006, Beynon *et al.* 2007, Beynon 2008, Gossop & Moos 2008).

### Health status, quality of life and ageing

Older drug users described how their health had deteriorated with age, with some explaining this as a consequence of drug use and others as the impact of both drug use and ageing. Decreased health status, physical and mental, is a feature of older populations and may be related to ageing and other factors (Bowling 2005), but remains to be extensively investigated in older people who continue to use drugs later in life. It is known that drug use and methods of administration, such as injecting and using shared syringes and needles, can cause serious infectious diseases such as hepatitis C (WHO 2000, Hser *et al.* 2004, De *et al.* 2008, HPA 2008) and other acute and chronic conditions such as deep vein thrombosis (Woodburn & Murie 1996), obstructive airways disease (Hser *et al.* 2004) and pneumonia (Beynon & McVeigh 2007). Psychosocial impacts of drug abuse in younger people can include disruptive, ant-social and aggressive behaviours (National Institute on Drug Abuse 2003). Depression and anxiety are more prevalent in older people (Age Concern 2007) and our findings indicate that older drug users experience substantial impact on their mental health and wellbeing, with depression, anxiety, aggression, disturbed sleep and phobia being common features. Chronic drug use could have accelerated the impact of ageing on their health status compared to other cohorts. Diminished quality of life was a feature for most of those interviewed, and was a consequence of drug use for their health, wellbeing, relationships, lifestyle, where they lived and the directions their lives had taken. Impaired quality of life as a consequence of ageing is the focus of much research (Ageing & Society 2004, Walker & Hagan Hennessey 2004, Bowling 2005), with UK policy and interventions aimed at maintaining the quality of life and social inclusion of older people (DH 2006, Social Exclusion Unit 2006, Windle *et al.* 2008, Gray 2009).

Similarly, the older people interviewed had accelerated or earlier life experiences of death and dying, through loss and deaths of relatives and other drug users, as well as confronting their own potential early mortality. Death and dying are features of the life course and of ageing and older age in particular (Tomer 2000), but for this population featured earlier in their lives (Bargagli *et al.* 2006). Reminiscence and life review are also features of ageing, and some of those interviewed reviewed their lives in context of their drug use and how their lives might have been different if they had not used drugs. A common theme was their survival in the context of their own drug use, overdoses, and the deaths of drug-using contemporaries, and their resilience. Reflections on survival and resilience form part of life review in older age (Bornat 1994), and are even more poignant for this population. A few people interviewed expressed wishes and plans for the future, in particular those who were abstinent of drugs or planned detoxification and future abstinence, compared to those who continued to use methadone and heroin, saw no change to their circumstances or had an escalating drug problem. These findings illustrate that older people with a history of continued or problematic drug use, including illicit drug use, are a particularly vulnerable group.

### Relationships, social networks, drug use and ageing

Most older people interviewed lived alone, were single or divorced and had a history of divorce, chaotic or fractured relationships, which in addition to ageing meant that they were a vulnerable social group within their communities. Three men described having a male friend who acted as carers, two living with them and one next door. All were themselves drug users, which was how they had met each other. In older ageing cohorts it is usual for women to undertake the role of carer (Arber 1998, Phillips 2000), and the gender difference here may reflect the breakdown or non-existence of conventional family and gender relationships or wider social change in kin-based relationships and caring (Pahl & Spencer 2004, Pahl & Pevalin 2005). Breakdown of relationships is associated with drug use (Crystal 1992, Velleman *et al.* 1998, 2003, Levy & Anderson 2005, Myerson 2009); indeed, the people interviewed described how they did not have ‘real friends’ but only ‘drug buddies’ or fellow ‘users’. Only a minority described lasting supportive relationships with key family members who were aware of their drug history and were accepting of their situation, while other family relationships with spouses, children, parents and siblings, for others had dissolved.

Most people interviewed described early, and in some cases extensive, experiences of the death or dying of people close to them, either family or drug users. This appeared to have been an accelerated experience for this population compared to older non-drug using people who experience such loss in later years. These particular experiences of ageing drug users regarding relationships and social networks can result in increased vulnerability, social isolation and lack of social capital, which is a concern for all older populations (Gray 2009).

### Ageing, older drug users and service use

The population of older people continuing to use illicit drugs is increasing, and they will require access to support services (Phillips & Katz 2001, Boeri 2004, Beynon *et al.* 2007, Gossop & Moos 2008). The older people in this study were receiving treatment and support for their drug addiction from drug services provided by the voluntary sector, about which they were positive. They had also required treatment as hospital inpatients or in primary health care settings, and described some instances of care that were prejudicial. However, there were also instances where positive attitudes and care had been received. This clearly demonstrates the importance of nurses and healthcare staff having non-judgemental attitudes and knowledge of drug use and addictions. The increasing age of drug users may mean that services should be tailored to meet their needs as they are a vulnerable group. The man interviewed who described how he had nothing in common with the younger generation of drug users with whom he attended group sessions at the drug treatment service clearly illustrates this. Another man preferred individual consultation and support as opposed to group sessions, which also indicates the need for tailored services in particular for older people, and that no single approach suits all (Crome & Bloor 2005a, 2005b, 2006). Older drug users have a lifetime of experience and could have an important role to play in health promotion and the prevention of drug use in younger populations. Involvement of service users in mental health service development and delivery is gaining recognition as an important aspect of provision (DH 2000, Ross *et al.* 2005, Age Concern 2007).

## Conclusion

Older people who continue to use drugs and require the support of services are emerging as an important but relatively under-researched international population. Older people who continue to use drugs are a vulnerable group, having impaired health status, chronic conditions, particular health needs and poorer quality of life due to their drug use, addiction and life experiences. Their relationships, social networks and support were also more restricted due to their lifestyles, and added to their vulnerability. Living alone and their accommodation also made them more susceptible to social isolation. All these factors need to be taken into consideration by nurses when planning, co-ordinating or providing care and treatment. Further research into the specific needs of this ageing population is warranted, and services should be developed to meet these needs.
